# Understanding the structure of the first atomic contact in gold

**DOI:** 10.1186/1556-276X-8-257

**Published:** 2013-05-29

**Authors:** Carlos Sabater, María José Caturla, Juan José Palacios, Carlos Untiedt

**Affiliations:** 1 Departmento de Física Aplicada, Universidad de Alicante, Carretera San Vicente del Raspeig, Alicante 03690, Spain; 2 Departamento de Física de la Materia Condensada, Universidad Autónoma de Madrid, Madrid 24105, Spain

**Keywords:** Electronic transport, Atomic size contacts, Mechanical annealing, Jump-to-contact phenomena, Jump-out-of-contact phenomena, Molecular dynamics simulations, *Ab initio*, DFT

## Abstract

We have studied experimentally jump-to-contact (JC) and jump-out-of-contact (JOC) phenomena in gold electrodes. JC can be observed at first contact when two metals approach each other, while JOC occurs in the last contact before breaking. When the indentation depth between the electrodes is limited to a certain value of conductance, a highly reproducible behaviour in the evolution of the conductance can be obtained for hundreds of cycles of formation and rupture. Molecular dynamics simulations of this process show how the two metallic electrodes are shaped into tips of a well-defined crystallographic structure formed through a mechanical annealing mechanism. We report a detailed analysis of the atomic configurations obtained before contact and rupture of these stable structures and obtained their conductance using first-principles quantum transport calculations. These results help us understand the values of conductance obtained experimentally in the JC and JOC phenomena and improve our understanding of atomic-sized contacts and the evolution of their structural characteristics.

## Background

Metallic atomic-sized contacts can be created by scanning tunneling microscopy (STM) [1,2] or by mechanically controlled break junctions [1,3]. In such nanocontacts, the electrical conductance is closely related to their minimum cross section. Therefore, by recording the conductance while the electrodes are displaced with respect to each other (traces of conductance), one can infer the atomic structure of these contacts. However, to understand the structures formed at the contact, it is necessary to make use of theoretical models. Landman et al. [4] pioneered the use of molecular dynamics (MD) simulations to follow the variation of the minimum cross section during the process of stretching a nanocontact. Later, Untiedt et al. [5], by experimentally studying the jump-to-contact (JC) phenomena in gold and combining MD and electronic transport calculations, were able to identify the formation of three basic structures before contact between the two electrodes, although a limited analysis on the conductance values was presented there.

Trouwborst et al. [6] have also studied the phenomena of JC and JOC using indentation loops where the maximum conductance was limited to 1*G*_0_, where $G_{0}=2\frac{e^{2}}{h}$ (quantum of conductance). These experiments showed that the elasticity of the two electrodes is one of the relevant parameters to explain these phenomena. Despite these, presently, there is not a unique picture that correlates the experiments with the MD and transport calculations regarding the different atomic structures that can be found at the contact.

On the other hand, experiments, together with molecular dynamics and electronic transport calculations based on density functional theory, show how very stable structures can be obtained by repeated indentation. This has been described as a mechanical annealing phenomenon [7]. Limiting the maximum conductance value (5*G*_0_ for gold) in the process of formation and rupture of a nanocontact leads to reproducible and atomically sharp pyramidal electrodes. This technique has recently been used by other authors [8] to prepare tips *in situ* for low-temperature STM.

In this paper, we show experimental results of the JC and JOC phenomena for gold that are analyzed simultaneously. We study the most probable configurations before the formation and breaking of nanocontacts with pyramidal form obtained from MD simulations emulating the process of mechanical annealing. As found earlier [5], the contacts can be classified into monomer, dimer and double contact. In order to correlate with the experimentally obtained conductance values, we calculated the conductance of these structures using first-principles quantum transport models.

## Methods

**Figure 1:**
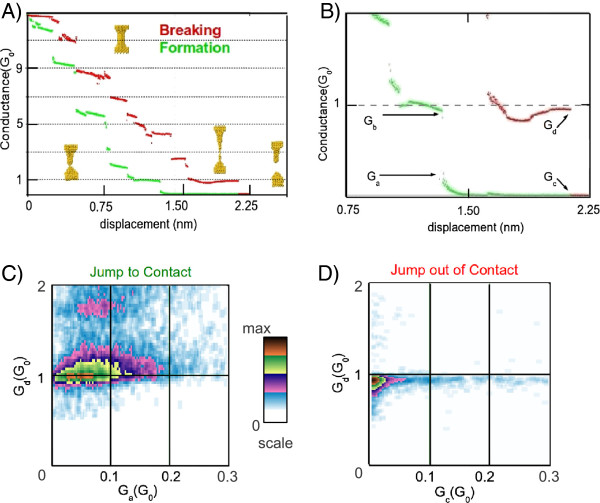
**How to build a density plot.** (**A**) On the top left-hand side, we show a typical trace of conductance of gold at 4 K during the process of breaking (red) and forming (green) a contact. (**B**) The top right-hand side is a zoom near 1*G*_0_ to define the values before and after the JC, *G*_*a*_ and *G*_*b*_, and JOC, *G*_*c*_ and *G*_*d*_. (**C, D**) The bottom figures show colour density plots where dark colours represent those values of conductance that appear more frequently (left for JC and right for JOC).

We have used an STM, where the tip and sample were two gold electrodes with 99.999*%* purity. The experiments were done at 4.2 K and cryogenic vacuum atmosphere. In order to obtain the conductance of the contacts, the electrical current was measured while applying a 100-mV constant bias voltage between the gold structures. Figure 1A shows traces of conductance in a gold nanocontact, measured in units of *G*_0_ during the process of formation (red) and rupture (green). Insets show some snapshots from our molecular dynamics simulations. These correspond to the initial structure (top figure) and the final structures before breaking (bottom right) and just after contact formation (bottom left). Figure 1B is a zoomed area around 1*G*_0_ of Figure 1A, where the phenomena of JC and JOC can be clearly observed. In order to quantify the jump occurring in these two processes, we define two conductance values for JC (*G*_*a*_, *G*_*b*_) and two values for JOC (*G*_*c*_, *G*_*d*_). These values correspond to the conductance values before and after the jump. We have performed thousands of indentations and recorded the values of these points. Representing *G*_*b*_ vs *G*_*a*_ for the JC case and *G*_*d*_ vs *G*_*c*_ for JOC, we can obtain a colour density plot as shown in Figure 1C for JC and in Figure 1D for JOC. Lighter colours are less probable values than darker colours.

To emulate the movement of the STM and simulate the tip and surface that are annealed mechanically, we used MD simulations with embedded atom potentials. Density function theory (DFT)-based calculations are performed to obtain the electronic transport in the simulated structures [9]. For the MD simulations, we have selected an embedded atom potential [10] because elasticity of the electrodes seems to be one of the key parameters in the processes to be studied [6], and these empirical potentials are fitted to reproduce the experimental elastic properties of bulk materials.

Furthermore, the computational cost with this simulation method is low, which makes it an appealing tool since we need to simulate tens of these cycles of breaking and formation of the nanocontact.

**Figure 2:**
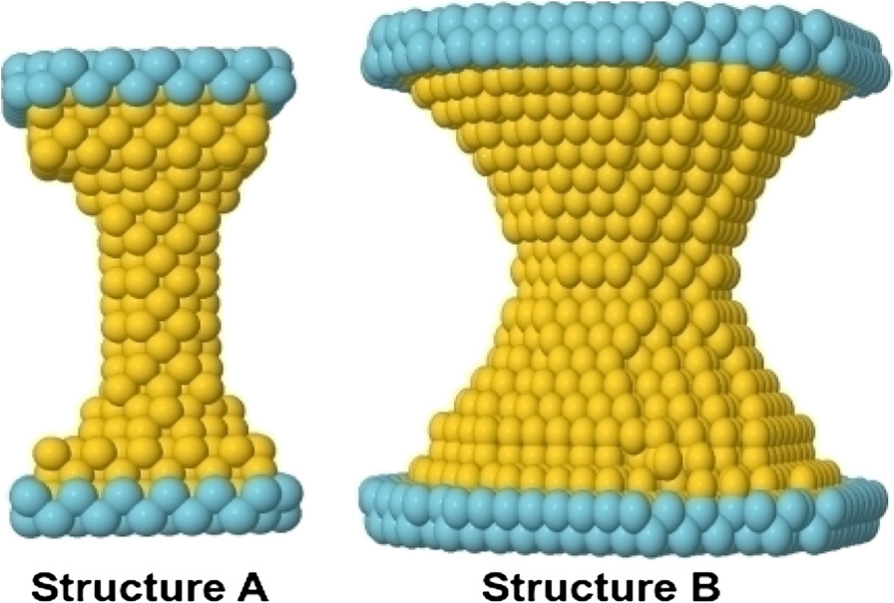
**Structure and configurations of contacts.** The two initial configurations used in the MD simulations are shown: **structure A**, long and narrow contact and **structure B**, short and wide contact.

**Figure 3:**
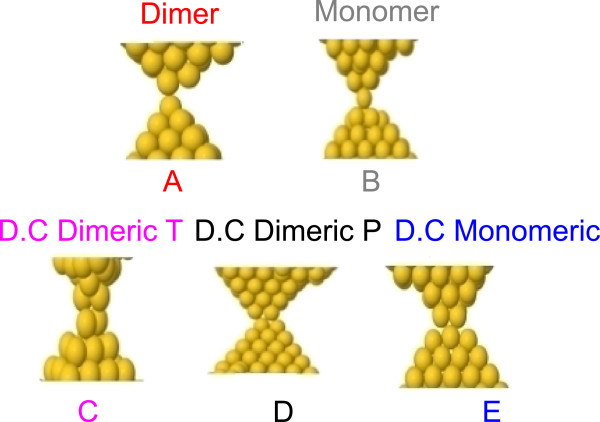
**Structures are the point of contact or before breaking from MD simulations.** Representative configurations obtained from MD simulations right before contact or right before breaking are shown. (**A**) dimer, (**B**) monomer, (**C**) double contact dimeric transversal, (**D**) double contact dimeric parallel and (**E**) double contact monomeric.

Using MD, we have analyzed the same structures described in detail in another study [7], but now, we focused on the type of contact formed. The two initial configurations of the nanocontacts are shown in Figure 2. Structure A is built with 525 gold atoms. This initial structure is stretched until the contact is broken by displacing the two top and bottom atom layers (represented in blue in the figure). After breaking, the direction of the displacement of these layers is reversed so that the two sides are brought together until contact. The temperature in the simulations is 4.2 K. In this case, the temperature is scaled in every cycle of breaking and formation of the contact. The indentation process continues until the minimum cross section formed has 15 atoms, and then the whole cycle starts again, breaking and forming the contact for a total of 20 cycles (see movie1 of supplementary material in reference [7]). The second structure studied (structure B) is shown in Figure 2; it is composed of 2,804 gold atoms. In this case, the indentation is limited to cross sections of 25 and 15 atoms (movie2 and movie3 at supplementary material on reference [7]). The temperature here is kept constant and is equal to 4.2 K during the whole simulation, which was done by scaling the velocities of all atoms every time step (every femtosecond). The strain rates applied are between 10^8^ and 10^10^ s ^−1^, which are typical of MD simulations [11]. Note that the ratio of length of the contact to the minimum cross section is very different in these two structures (5 for structure A and 2 for structure B), therefore exploring a system with a long and narrow constriction and another of a short and wide nanowire. As shown previously [7], structure A reaches a stable configuration formed by two pyramidal tips after repeated indentations. This configuration is formed after cycle 11, and it remains stable for the following 9 cycles. In each of these cycles, although the pyramidal shape remains, there are differences in the atomic configurations right at the contact, as shown in Figure 3. These are the configurations we study and describe in this paper in detail. For the case of structure B, because of the initial shape, the formation of the two pyramidal tips occurs from the very first cycle, and again, only differences are observed in the very last atomic configuration forming the contact. We have performed electronic transport calculations based on DFT [9,12] for both structures A and B. These calculations have been carried out with the help of our code ANT.G, which is part of the package ALACANT [9] and implements the non-equilibrium Green’s function formalism as a module to the popular code GAUSSIAN09 [12]. Here, due to the large number of atoms, we have employed a very basic basis set consisting only of one 6*s* orbital and one electron, the remaining 78 electrons being part of the pseudopotential.

## Results and discussion

Experimental results of the JC and JOC in gold are shown in Figure 1 and Table 1. Figure 1C,D shows the colour density plots obtained for gold when representing *G*_*b*_ vs *G*_*a*_ for the case of JC and *G*_*d*_ and *G*_*c*_ for the case of JOC. Note the presence of two very distinct areas in the JC plot corresponding to configurations with a high probability. In the case of JOC, we can distinguish clearly one area of high probability. More details about these experiments are presented in reference [5]. For clarity, we included in Table 1 those pairs of conductance that appear more frequently in the experimental measurements. We should mention that for all traces studied in gold, the phenomena of JC or JOC are always observed, unlike in other metals [5]. For JC, we observed three pairs of values that occur with higher frequency which we named as maxima 1, 2 and 3. In JC, maxima 1 and 2 correspond to jumping from a value of 0.01*G*_0_ to a value of 0.94*G*_0_ and from a value of 0.05*G*_0_ to 0.98*G*_0_. These two peaks are easily observed in Figure 1C as one large area of high probability. The last maximum corresponds to a jump from 0.09*G*_0_ to 1.77*G*_0_, which is the second spot shown in Figure 1C. On the other hand, on breaking the nanocontact, only two maxima have been identified: one where the contact breaks for conductance values of 0.92*G*_0_, which is clearly seen in Figure 1D, and another one when it breaks at conductance values of 1.60*G*_0_, which appears very faint in the figure. Note that these two values are close to those obtained for the first and third maxima in the JC case.

**Table 1:** Experimental values of conductance that appear more frequently in the case JC and JOC

**Pairs of values obtained in the density plots in Figure**1			
**Phenomena**	**Maximum 1**	**Maximum 2**	**Maximum 3**
	**(*G***_***a***_**,*G***_***b***_**)*G***_**0**_	**(*G***_***a***_**,*G***_***b***_**)*G***_**0**_	**(*G***_***a***_**,*G***_***b***_**)*G***_**0**_
JC	(0.03,0.94)	(0.05,0.98)	(0.09,1.77)
JOC	(0.01,0.92)	-	(0.01, 1.60)

Pairs of values (***G***_***a***_, ***G***_***b***_) and (***G***_***c***_, ***G***_***d***_) for JC and JC-JOC, respectively, which appear more frequently in the density plot of Figure 1.

As mentioned, we make use of molecular dynamics simulations and DFT calculations of conductance to understand these experimental measurements and observations. Figure 2 shows the two structures studied using MD, as described earlier. In Figure 3, we show some snapshots of the configurations found just after the contact between the two tips and just before breaking a nanocontact. Three basic atomic structures are found: a monomer (Figure 3A), a dimer (Figure 3B) and a double contact (D.C.) (Figure 3C,D,E). For the case of a double contact, we have identified different geometries, three of which are shown in this figure. We introduce, for the first time, the concept of a double dimeric (Figure 3C,D) and monomeric (Figure 3E) contact. We define a double dimeric contact as the one where the contact is between two atoms facing two other atoms, while we define a double monomeric contact as a contact where two atoms are contacting each other. Another interesting point is that for the double dimeric contact, we have identified two possible structures: one where two atoms are perpendicular to the other two (Figure 3C), which we call transversal configuration (D.C. Dimeric T), and one where two atoms are parallel to the other two (Figure 3D), which we call parallel configuration (D.C. Dimeric P).

Table 2 shows the probability of finding a monomer, a dimer or a double contact (all possible configurations for D.C.) in the MD simulations right before contact and right after contact for the two initial structures and different indentations. Note the limited statistics in these results since only 10 cycles have been computed for the first structure and 9 cycles for the second one. Nevertheless, we can see some interesting results. For the case of structure A, with a large ratio of length to minimum cross section, we observe that the most probable configuration both at JC and at JOC is a dimer. The monomer and the double contact have similar probabilities. This result is in agreement with reference [13]. The situation for the structure B, with a small ratio of length to minimum cross section, is significantly different. In this case, when the indentation between the two tips is limited to 15 atoms in cross section, the configuration at the contact is the same in all cycles, a double contact, although we observe the formation of the different double contacts described in Figure 3C,D,E. Clearly, very stable pyramidal structures are formed in this case. The robustness of the tip imposes the repetition of a certain kind of structure. When the indentation between the two tips increases to a value of 25 atoms in cross section, we should note that the traces do not repeat between cycles, and therefore, different structures are formed. In this case, for JC, the double contact is still predominant, while for JOC, the probabilities have the same trend as in structure A (dimer being the most probable).

**Table 2:** MD results of first or last contact (JC/JOC) type in structures A and B annealed mechanically

**Percentage of cases of type monomer, dimer and D.C.**			
**Phenomena**	**Monomer**	**Dimer**	**Double contact**
JC structure A 15 inden	20	60	20
JOC structure A 15 inden	30	60	10
JC structure B 15 inden	0	0	100
JOC structure B 15 inden	0	0	100
JC structure B 25 inden	22	0	78
JOC structure B 25 inden	22	56	22

This table contains the probabilities of finding a dimer, monomer or double contact (D.C.) in the MD simulations for the two structures (A and B) studied during the formation or the breaking of the contact and for different indentation (inden) values (15 atoms or 25 atoms in the minimum cross section).

In order to correlate the results from molecular dynamics to the experimental measurements, it is necessary to calculate the conductance of these atomic structures. Table 3 shows the values of conductance obtained from electronic transport calculations based on DFT for the typical first or last contacts proposed: monomer, dimer and double contacts. The table includes the values of conductance obtained with their standard deviation. We can observe that the monomer values of conductance are in the range 1.20*G*_0_ to 0.76*G*_0_, with an average value of 0.97*G*_0_. That is because, during the process of rupture and formation, the monomer can be localized closer or further away from the rest of the contact. Another important factor that can change the conductance of a monomer is the total number of neighboring atoms to the central atom in the contact, which can be different while remaining a monomer structure. Both factors are responsible for the spread in the conductance values of a monomer. On the other hand, the deviations in the conductance values for dimer or double contact structures are significantly smaller, around 0.07*G*_0_ and 0.02*G*_0_, respectively, the average conductance value being 0.92*G*_0_ for a dimer and 1.73*G*_0_ for a double contact. These results indicate that, on average, dimers and monomers have similar values of conductance while double contacts have significantly larger conductance values. It seems clear then that the maxima obtained experimentally for JC and JOC, with conductance values of 1.77*G*_0_ and 1.6*G*_0_, respectively (maximum 3 for JC and maximum 2 for JOC in Table 1), correspond to the formation of a double contact. The results for the other maxima obtained experimentally are not so clear since the average conductance values obtained for a monomer and a dimer in the calculations are very similar. This seems to indicate that the two first maxima obtained experimentally in the JC must correspond to configurations in a dimer and in a monomer geometry. According to MD simulations, the most likely configuration both in JC and JOC is a dimer (except in special cases of very stable tips), although monomers can also be formed.

**Table 3:** Electronic conductance calculated by DFT on typical contacts obtained from MD structures

**Structure and value of conductance*G***_**0**_			
**Metal**	**Dimer**	**Monomer**	**Double contact**
Au	0.92 ± 0.07	0.97 ± 0.15	1.73 ± 0.02

**Figure 4:**
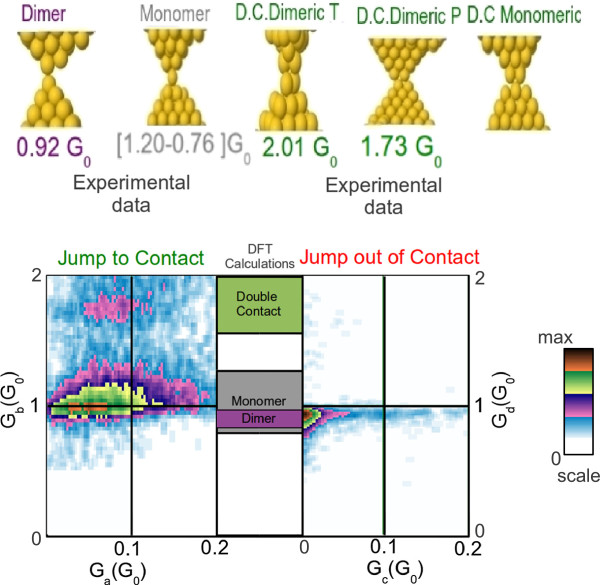
**JC and JOC density plots together with conductance calculations of different geometries of the contact.** Inside the experimental density plots, we have marked the average conductance values after or before the jump as obtained from DFT electronic transport calculations with their deviations.

Figure 4 combines the experimental results with the calculations of conductance in order to identify the experimental values of JC and JOC with structures detected by MD and calculated by DFT. The center column between the experimental density plots of JC and JOC indicates the average value of conductance obtained from the simulations for each geometry (double contact, monomer and dimer). The thickness of the rectangles around each geometry indicates the standard deviation. It is clear from this plot that the top high frequency events in the density plots corresponds to a double contact and the bottom high frequency events corresponds to monomer and dimer configurations. Although, as we mentioned, it is difficult to distinguish the monomer and dimer using our theoretical model, we can see that the average of conductance of monomers is above the one of the dimers. If we add to this that we would expect a higher tunnel conductance (on average) prior to the formation of a monomer, we can label maxima 1 and 2 as dimer and monomer, respectively.

## Conclusions

Experiments of JC and JOC show that certain structures are more likely to occur than others. This depends on the metal and on the process of breaking/formation and the type of structure at the electrodes. Simulations and calculations (MD and DFT) of these experiments show that three basic atomic structures are formed at the contact: monomers, dimers and double contacts. We have identified within the double contact structure several different atomic arrangements that we named double dimeric contact (parallel and perpendicular), and double monomeric contact. According to DFT electronic transport calculations, double contacts have an average value of conductance of 1.73*G*_0_, which correlates very well with one of the peaks observed experimentally both for JC and for JOC. This configuration is also obtained in JC and JOC from the MD simulations and, for some very stable tips, is the dominant configuration. Monomers and dimers, however, are difficult to distinguish from the simulations since their average conductance values are very similar (0.97*G*_0_ and 0.92*G*_0_, respectively). In the case of JOC, these two peaks cannot be resolved. Interestingly, the conductance values are somehow lower than in the case of JC, which could indicate the most likely formation of stretched contacts.

## Competing interests

The authors declare that they have no competing interests.

## Authors’ contributions

CS wrote the manuscript and did MD simulations and DFT calculations. CU and CS performed the experiments. MJC and JJP supervised the MD and DFT calculations. All the authors have participated in the outline of this research, in the bibliographical study and revised the manuscript. All authors read and approved the final manuscript.
